# Actuating Voltage Waveform Optimization of Piezoelectric Inkjet Printhead for Suppression of Residual Vibrations

**DOI:** 10.3390/mi11100900

**Published:** 2020-09-29

**Authors:** Muhammad Ali Shah, Duck-Gyu Lee, Bo Yeon Lee, Nam Woon Kim, Hyojin An, Shin Hur

**Affiliations:** 1 Korea Institute of Machinery and Materials, Daejeon 34103, Korea; ali@kimm.re.kr (M.A.S.); educk9@kimm.re.kr (D.-G.L.); bylee@kimm.re.kr (B.Y.L.); nwkim@kimm.re.kr (N.W.K.); kky5523@kimm.re.kr (H.A.); 2 Department of Nano-Mechatronics, University of Science and Technology, Daejeon 34113, Korea

**Keywords:** residual vibrations, optimized waveform, piezoelectric inkjet printhead

## Abstract

After a piezoelectric inkjet printhead jets the first droplet, the actuating membrane still vibrates, creating residual vibrations in the ink channel, which can degrade the inkjet printhead performance. For suppressing these vibrations, an optimized actuating voltage waveform with two pulses must be obtained, of which the first pulse is used for jetting and the second pulse is used to suppress the residual vibrations. In this study, the pressure history within the ink channel of a recirculating piezoelectric inkjet printhead was first acquired using lumped element modeling. Then, for suppressing residual vibrations, a bipolar voltage waveform was optimized via analysis of the tuning time ($t_{t}$ ), dwell time ($t_{d2}$), rising time ($t_{r2}$), falling time ($t_{f2}$), and voltage amplitude of the second pulse. Two voltage waveforms, Waveform 01 and Waveform 02, were optimized thereafter. In Waveform 01, $t_{t}=2\;\mu s,\;t_{d2}=2\;\mu s$, and $t_{r2}$ and $t_{f2}=1\;\mu s$ were finalized as the optimal parameters; in the case of another waveform, the optimal parameters of $t_{d2}$, $t_{r2}$, and $t_{f2}$ were found to be 4, 1, and 1 μs, respectively. The optimal voltage amplitude of the second pulse was found to be 1/3 the amplitude of the first pulse. On the basis of our analysis, the tuning time in Waveform 01 is the most sensitive parameter, and the performance yielded is even poorer than that yielded by standard waveform, if not optimized. Therefore, the other waveform is recommended for the suppression of residual vibrations.

## 1. Introduction

Continuous streams of developments in the field of printing technology were performed by the researchers over the past few decades. Several methods i.e., needle-based printing [1], Acoustophoretic printing [2], piezoelectric and thermal inkjet printheads-based printing [3,4], electrohydordyanic jet printing [5], laser-based printing [6], surface acoustic waves printing [7] and drop impact printing [8] have been demonstrated in the literature to get the ultimate goal of precise printing on its challenging pathway. Among these methods, piezoelectric inkjet prinheads based printing are commonly used due to its advantage of allowing precisely controlled drop volume by varying the actuating voltage waveform applied to the piezoelectric membrane.

The working principles of a piezoelectric inkjet printhead involve two mechanisms: driving and ejection. The ejection of the ink droplet is achieved via the driving mechanism. During the driving stage, the piezoelectric membrane is deformed via excitation using a voltage waveform, as a result of which pressure is generated in the pressure chamber that drives the ink into the nozzle, causing the ejection of droplets at the nozzle exit. Typically, a single-pulse voltage waveform, referred to here as a standard waveform, is used to drive the piezoelectric membrane. However, after the ejection of the first droplet using standard waveform, pressure waves still exist in the pressure chamber. These unwanted pressure waves are referred to as residual vibrations. These residual vibrations take several microseconds to decay, which affect the velocity and volume of the second droplet to be fired, thereby affecting the printing quality [9,10,11]. Due to these residual vibrations, satellite droplets are also formed that can fall on an undesired position on a substrate [12]. To suppress these residual vibrations, a two-pulse voltage waveform, either unipolar [12,13] or bi-polar [14,15], is reported in the literature. In the two-pulse voltage waveform, the first pulse is used for jetting purposes and the second pulse is used to suppress the residual vibrations. The advantage of using a bipolar waveform is that the residual vibrations can be damped earlier compared with unipolar waveforms [10].

The optimization of voltage waveforms with bipolar pulses has been a topic of interest in the research community for precise impinging of ink droplets onto various substrates. Rising and falling times for the first pulse of less than 3 μs are sufficient for good drop ejection [16]. However, the dwell time also needs to be tuned. The optimal dwell time can either be determined using acoustic propagation and reflection theory [16] or numerical simulations [17]. These two approaches can be used only to determine the optimized dwell time of the first pulse. As the second pulse is related to suppression of residual vibrations, a model-based or experimental approach is needed to determine its optimal parameters. For this purpose, the pressure or velocity history of the meniscus or pressure chamber is needed. Numerical calculations and simulations [17,18], narrow channel theory [19,20], model-based [10,21,22,23], and experimental [13,14] approaches have been used to acquire the pressure and velocity histories at the pressure chamber and nozzle. Among these methods, the experimental or model-based approach can be used to determine the optimal parameters of the bipolar voltage waveform. In [14], the authors used an experimental approach to measure the meniscus motion to optimize the voltage waveform.

The experimental approach provides limited access to the pressure and velocity histories. More information is needed to better understand the interior of the inkjet printhead. Therefore, special models for the development of new inkjet printheads are required, especially with respect to suppressing residual vibrations. On the basis of these models, the interior of the printhead can be easily accessed. Once the pressure or velocity histories have been determined, the optimized voltage waveform can be easily obtained simply via tuning of its parameters.

The authors of [10,23] used a model-based approach to obtain the optimal voltage waveform with respect to suppressing residual vibrations. However, detailed analysis of the suppression of residual vibrations is still missing in these previously published research articles. Moreover, the designs used in these articles are non-recirculating. Problems associated with non-recirculating designs include pigment sedimentation, drying of the ink in the nozzles, and nozzle blockage [15,24]. Some designs with ink recirculation have been reported in the literature to prevent these problems [25,26]; however, for a recirculating piezoelectric inkjet printhead, articles containing the optimization of voltage waveforms for the suppression of residual vibrations are missing in the literature to the best of the authors’ knowledge. Therefore, this topic needs to be explored. In this study, we used a lumped element model (LEM) to optimize the voltage waveform of a recirculating piezoelectric inkjet printhead for the suppression of residual vibrations.

## 2. Equivalent Circuit Model

**Figure 1:**
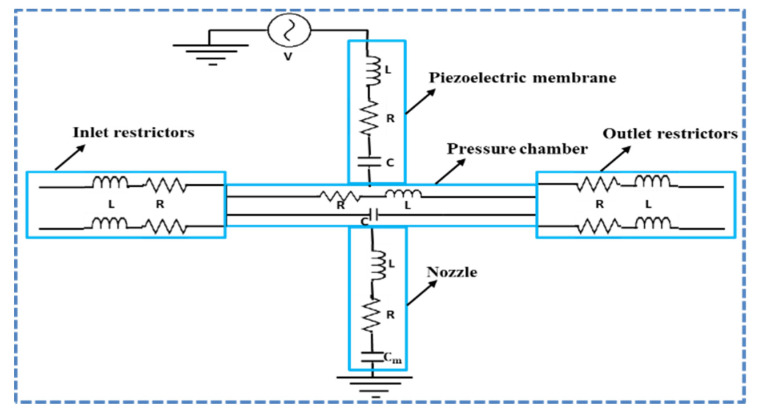
Equivalent circuit of a recirculating piezoelectric inkjet printhead.

**Figure 2:**
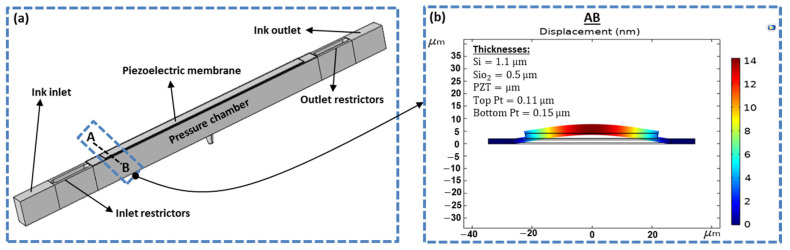
(**a**) Recirculating inkjet printhead; and (**b**) a displaced cross-sectional view of a piezoelectric membrane on the top of the pressure chamber.

The equivalent circuit of a recirculating piezoelectric inkjet printhead is shown in Figure 1, which includes the piezoelectric membrane, inlet and outlet restrictors, pressure chamber, and nozzle. For describing the behavior of the fluid in a lumped element model, three parameters, namely inductance (*L*), resistance (*R*), and capacitance (*C*), can be derived. *L*, *R*, and *C* are related to the fluid mass, viscous losses, and fluid energy storage. Similarly, in the case of an actuating piezoelectric membrane, *L* is related to inductance, *R* is the measure of mechanical loss of the piezoelectric membrane, and *C* is the mechanical compliance. The piezoelectric membrane is actuated through supply of an electric voltage. These electrical, mechanical, and fluidic domains are coupled together to form a two-port LEM. The coupling equations for a two-port model can be written as [27].

(1) $$\left\{\begin{array}{c} \Delta V\\ q \end{array}\right\}=\left[\begin{array}{cc} C_{a} & d_{a}\\ d_{a} & C_{e} \end{array}\right]\left\{\begin{array}{c} P\\ V \end{array}\right\},$$

where ΔV is the deformed volume with applied pressure P, q is the current charge on the piezoelectric electrodes, *V* is the applied voltage, $C_{a}=\Delta V/P$ is the acoustic compliance of the piezoelectric membrane at  V=0, $d_{a}=\Delta V/V$ is the effective acoustic piezoelectric coefficient at P=0, and $C_{e}$ is the electrical capacitance of the piezoelectric material, which can be determined experimentally or estimated using the following equation:

(2) $$C_{e}=\frac{\varepsilon_{r}\varepsilon_{0}A}{g},$$

where $\varepsilon_{r}$ and $\varepsilon_{0}$ are the dielectric constant of the piezoelectric material and the permittivity of free space, respectively; *A* is the area of the piezoelectric material; and *g* is the gap between the two electrodes. The deformed volume (ΔV) was calculated by integrating the displacement over the entire area of the piezoelectric plate. For the displacement estimation, COMSOL Multiphysics software (4.3b, COMSOL Inc., Stockholm, Sweden) was used. The two-dimensional deflected profile of the piezoelectric membrane with a displacement of 14 nm, which was pressurized at the interior surface, is shown in Figure 2b. Figure 2a shows the recirculating inkjet printhead with inlet and outlet restrictors, pressure chamber, and piezoelectric membrane. The thicknesses of silicon oxide (${\mathrm{Sio}}_{2}$), silicon (Si), top and bottom platinum (Pt) electrodes, and piezoelectric material lead zirconate titanate (PZT) are presented in Figure 2b.

Due to the surface tension formed by the interface between the air and fluid at the nozzle exit, a meniscus is generated. The capacitive term $C_{m}$ represents the fluid stored in the meniscus and is given by the following equation

(3) $$C_{m}=\frac{V_{men}}{P_{men}}=\frac{1/2\times \left(4\pi r_{n}{}^{3}\right)/3}{2\sigma /r_{n}}=\frac{\mu r_{n}{}^{4}}{3\sigma },$$

where $V_{men}$ is the half sphere volume of the fluid with the nozzle radius $r_{n}$, $P_{men}$ is the Laplace pressure, and σ is the coefficient of surface tension. Details of the two-port LEM of a recirculating piezoelectric inkjet printhead with the device dimensions, ink properties, derivation of differential equations, and mathematical equations for *R*, *L* and *C* of the actuating piezoelectric membrane and fluid can be found in our previous study [22].

## 3. Results and Discussion

**Figure 3:**
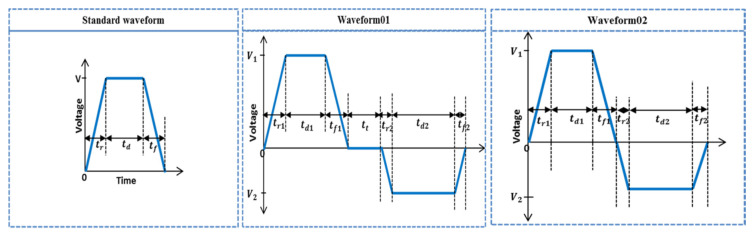
Analyzed waveforms in the present study.

For a recirculating piezoelectric inkjet printhead, a LEM was used in our previous study [22], and the results were validated by comparing it with numerical simulations. In our current study, we used the same model to optimize the voltage waveform for the suppression of residual vibrations. Three voltage waveforms, namely the standard waveform, Waveform 01, and Waveform 02, shown in Figure 3, were investigated, in which the standard waveform was optimized to obtain the maximum jetting pressure, while Waveform 01 and Waveform 02 were optimized for the suppression of residual vibrations generated by the standard waveform.

### 3.1. Standard Waveform Optimization

For a standard waveform, we considered the rising and falling times to be  1 μs, and the dwell time was optimized using acoustic wave propagation theory and our LEM approach. The optimum dwell time of  l/c was recommended in the acoustic waveform propagation and reflection theory presented by Bogy and Talke [16], where l is the length of the dispenser tube and c is the effective speed of sound. In [28], the optimum dwell time was found to be 2l/c. In another study, the authors concluded that the dwell time can be either  l/c or  2l/c [29]. The theoretical optimum dwell time is still uncertain. Therefore, we optimized the dwell time, both theoretically and using our LEM approach.

The effective speed of sound is reduced compared with the intrinsic speed of sound of the ink because of the piezoelectric membrane. The effective speed of sound is given by the following equation [21].

(4) $$c=\frac{c_{i}}{\sqrt{1+\frac{\Delta V}{P}.\frac{\rho c_{i}{}^{2}}{V_{0}}}},$$

where $c_{i}$ is the intrinsic speed of sound, ΔV is the deformed volume, P is the applied pressure, ρ is the density of the ink, and $V_{0}$ is the initial static volume of the pressure chamber. We used the approach presented in [21] to find the effective speed of sound, which was found to be 995.49 m/s. Considering the length of the dispenser tube to be  870 μm and using the formula  2l/c, the dwell time was found to be  1.747 μs≈2 μs.

**Figure 4:**
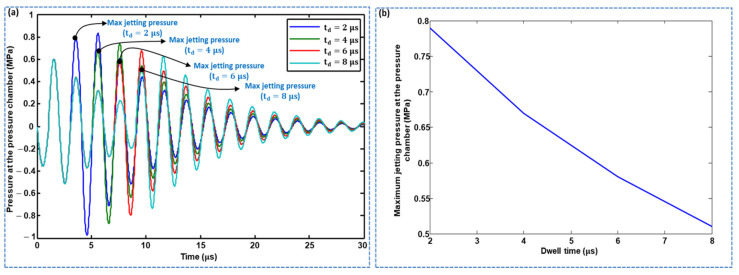
Effect of dwell time of the standard waveform on jetting pressure at the pressure chamber: (**a**) pressure profiles with various $t_{d}$, and (**b**) effect of dwell time on maximum jetting pressure.

**Figure 5:**
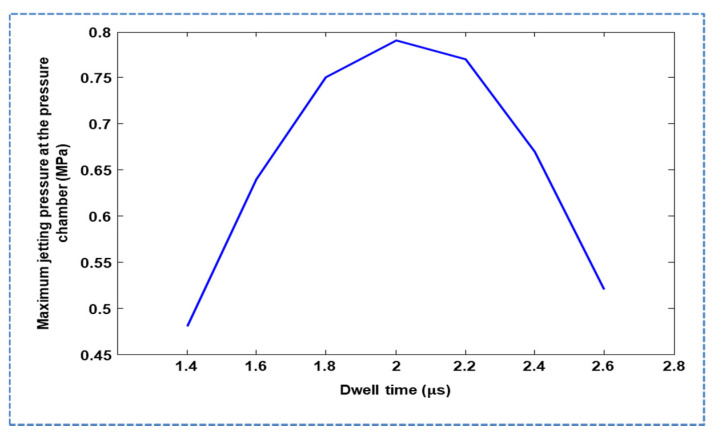
Effect of dwell time on jetting pressure at the pressure chamber with short time intervals.

**Figure 6:**
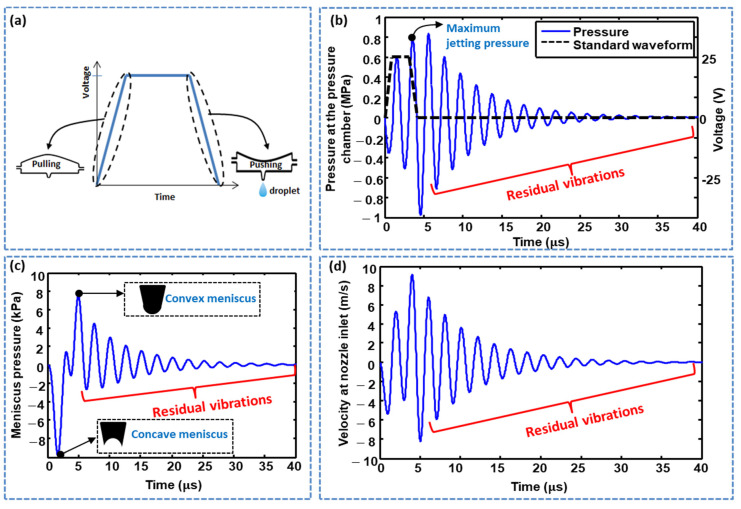
(**a**) Actuating mechanism of a piezoelectric inkjet printhead; (**b**) pressure profile at the pressure chamber; (**c**) meniscus pressure profile; and (**d**) velocity profile at the nozzle inlet generated by the standard voltage waveform.

The dwell time was also optimized using lumped modeling. Using LEM, the pressure history at the pressure chamber was acquired, and dwell times ($t_{d}$) of 2, 4, 6, and 8 μs. were considered. As shown in Figure 4a,b, the maximum jetting pressure decreased with increasing dwell time. The effect of dwell time on the maximum jetting pressure was also explored in the case of a short time difference, in which a $t_{d}$ of 2 μs resulted in the maximum pressure being yielded, as shown in Figure 5. The standard waveform was finalized with the parameters $\;t_{r}=1\;\mu s$, $\;t_{d}=2\;\mu s$, and $t_{f}=1\;\mu s$, and a pull–push actuation mechanism was used, that is, the piezoelectric membrane was pulled by the rising time and pushed by the falling time, where ink droplet ejection occurs at the pushing stage, as shown in Figure 6a. During the pulling stage, a negative pressure is generated in the pressure chamber, while, in the pushing stage, a positive pressure of approximately 0.8 MPa is generated, as shown in Figure 6b. Histories of meniscus pressure with a convex maximum pressure of 8 kPa and velocity with a maximum jetting velocity of approximately 9 m/s were also acquired, as shown in Figure 6c,d, respectively. A voltage amplitude of 25 V was used.

Although the optimized standard waveform can emit the ink droplet, there are residual vibrations after the ejection of the first droplet, as shown in Figure 6b–d. The next droplet properties will be different from that of the first one if these residual vibrations are not damped, which can affect the jetting process [9,10,11,12]. Through quick damping of these residual vibrations and bringing the ink channel to rest after the ejection of the first droplet, the maximum jetting frequency of the inkjet printhead can be increased. To suppress these residual vibrations, a waveform having two pulses must be employed. On the other hand, the droplet formation can be controlled by manipulating the voltage waveform [30].

### 3.2. Optimization of Waveform 01 and Waveform 02

The optimized standard waveform was used as the first pulse in the optimization of Waveform 01 and Waveform 02. The parameters of the second pulse, that is, the rising and falling times ($t_{r2}\;\;\mathrm{and}\;\;t_{f2}$), the dwell time ($t_{d2}$) of the second pulse of both waveforms, the tuning time ($t_{t}$) of Waveform 01, and the voltage amplitude of the second pulse are the most sensitive parameters in the case of residual vibrations, especially the tuning time ($t_{t}$). Therefore, these parameters need to be optimized. If these parameters are not optimized, the residual vibration amplitude can be amplified, and the jetting performance may be even worse than that obtained when using only the standard waveform. In all the results, the pressure profile of the standard waveform was compared with the pressure profiles of Waveform 01 and Waveform 02 to observe the suppression of residual vibrations by changing the parameters of the second pulse of these two waveforms.

#### 3.2.1. Waveform 01 Optimization

**Figure 7:**
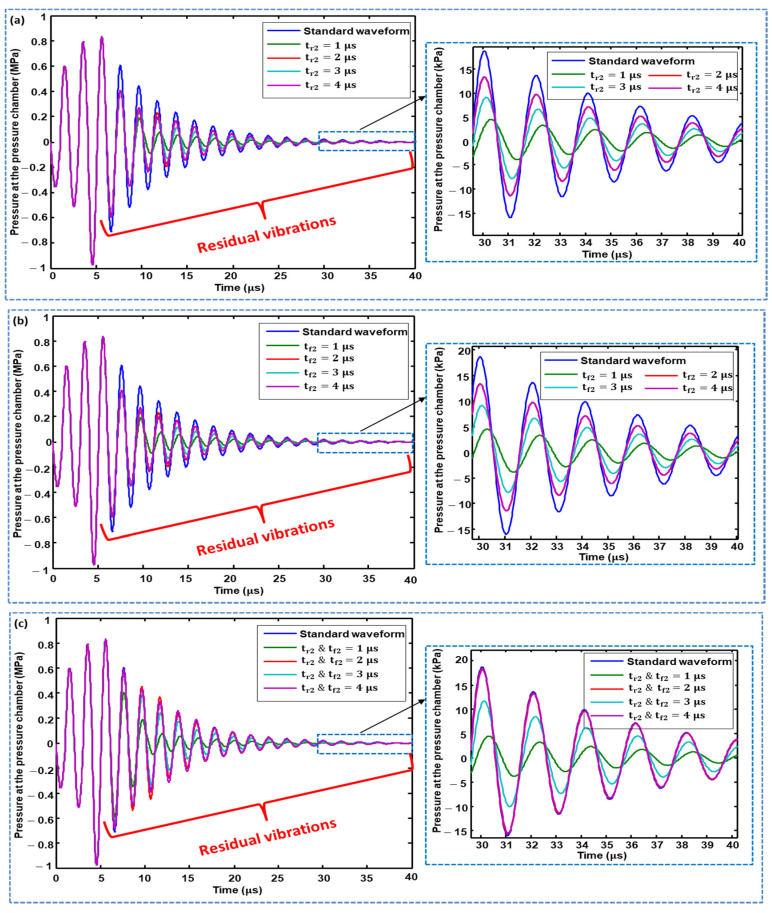
Effect of: (**a**) changing the rising time; (**b**) changing the falling time; and (**c**) simultaneously changing the rising and falling times of the second pulse of Waveform 01 on residual vibrations.

The effects of changing the rising time ($t_{r2}$), changing the falling time ($t_{f2}$), and simultaneously changing the rising and falling times of the second pulse of Waveform 01 on the residual vibrations were analyzed, and the optimal values for parameters $\;t_{r2}\;\mathrm{and}\;\;t_{f2}$ were finalized. $\;t_{r2}$ values of 1, 2, 3, and 4 μs suppressed the amplitudes of the residual vibrations compared with the standard waveform, where $\;t_{r2}=1\;\mu s$ gives the best performance, as shown in Figure 7a. The case with $\;t_{f2}$ is similar (see Figure 7b). When changing $\;t_{r2}\;\mathrm{and}\;t_{f2}$ simultaneously,$\;t_{r2},\;t_{f2}=1\;\mathrm{and}\;3\;\mu s$ suppressed residual vibrations, in which $\;t_{r2},\;t_{f2}=1\;\mu s\;$ demonstrated better performance, while that of $\;t_{r2},\;t_{f2}=2\;\mathrm{and}\;4\;\mu s$ had no effect on residual vibrations, as shown in Figure 7c. Based on these analyses, $\;t_{r2}\;\mathrm{and}\;\;t_{f2}=1\;\mu s$ was finalized as an optimal parameter.

**Figure 8:**
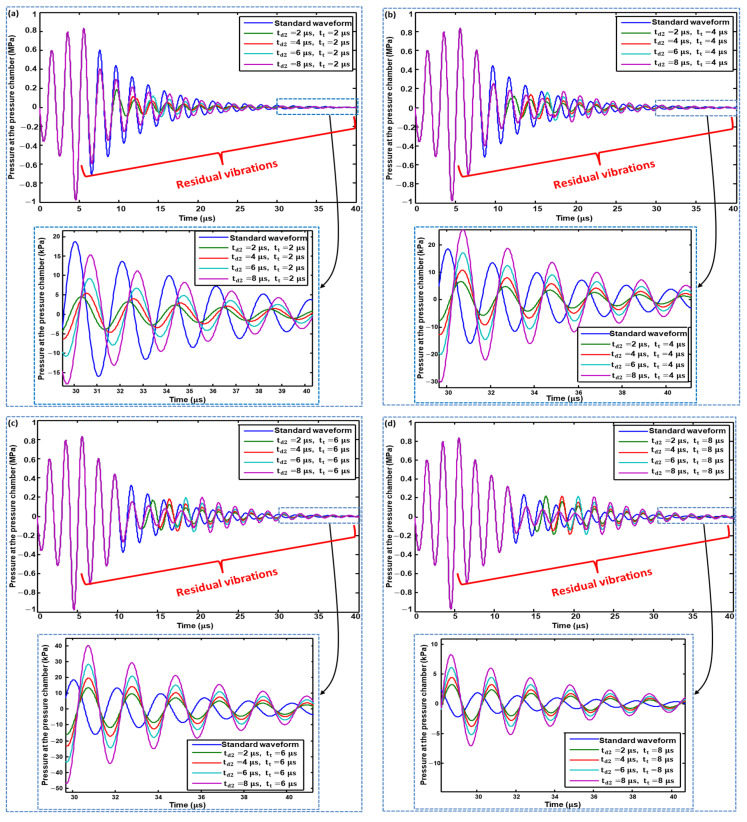
Effect of changing the dwell time of the second pulse of Waveform 01 on residual vibrations when tuning time is: (**a**) 2 μs; (**b**) 4 μs; (**c**) 6 μs; and (**d**) 8 μs.

In Figure 8a, the effect of changing $t_{d2}$ on the residual vibrations was analyzed by maintaining $t_{t}=2\;\mu s$ constantly. The amplitude of the residual vibrations on all the values of $t_{d2}$ is smaller if we compare it with the residual vibrations generated by the standard waveform. This means that the second pulse of the Waveform 01 successfully suppressed the residual vibrations, in which $t_{d2}=2\;\mu s$ gives better performance compared with 4, 6, and 8 μs. The same variation of $t_{d2}$ was explored with an increase in the tuning time,$\;t_{t}$, to 4 (Figure 8b), 6 (Figure 8c), and 8 μs (Figure 8d). With a $\;t_{t}$ of  4 μs, a $t_{d2}$ of 2 μs still suppressed the residual vibrations the most; however, a $t_{d2}$ of 8 μs yielded worse results than even the standard waveform, as shown in Figure 8b. Upon further increasing $\;t_{t}$ to 6 and  8 μs, all the values of $t_{d2}$ yield poorer performance than the standard waveform, as shown in Figure 8c,d.

The second pulse has no effect on the jetting pressure. The optimized parameters of Waveform 01 are presented in Table 1.

From this analysis, it was concluded that the time interval between the two pulses, that is, the tuning time, $\;t_{t}$ is a very sensitive parameter in the case of residual vibrations. It can lead to even poorer performance compared to that obtained when using the standard waveform if it is not optimized. It was also concluded that, through increasing the dwell time $\;t_{d2}$ of the second pulse, the performance became worse. In the case of changing $\;t_{r2}\;\mathrm{and}\;t_{f2}$, odd values led to better performance compared with even values.

#### 3.2.2. Waveform 02 Optimization

**Figure 9:**
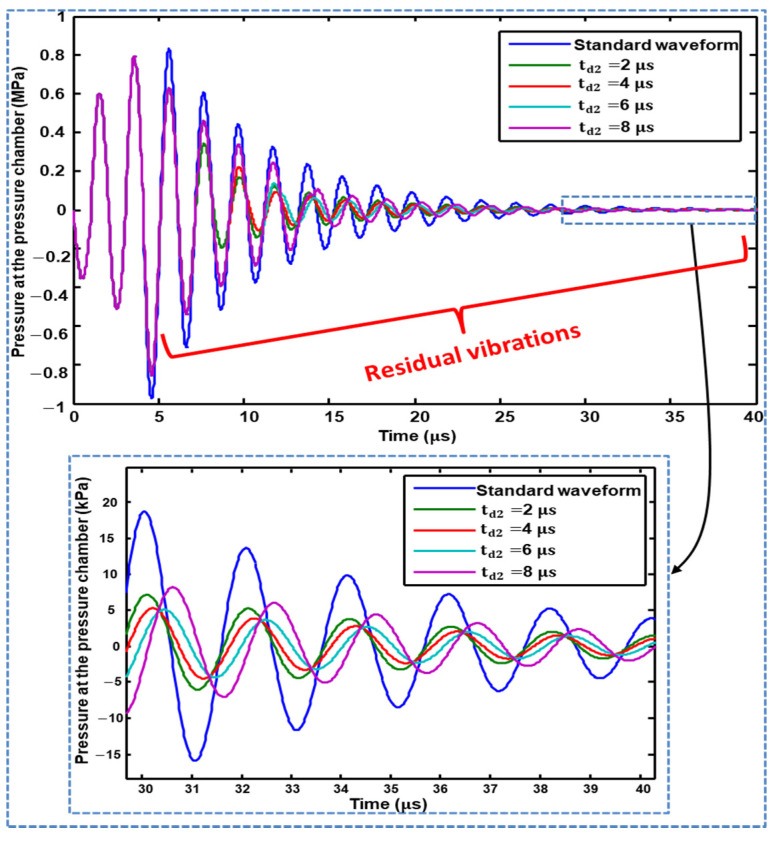
Effect of changing the dwell time of the second pulse of Waveform 02 on residual vibrations.

In the case of Waveform 02, the second pulse successfully suppressed the residual vibrations. The effect of changing the dwell time ($t_{d2}$) of the second pulse was compared with the standard waveform. Dwell time $\;t_{d2}=4\;\mu s$ gives the best results with respect to suppressing residual vibrations, as shown in Figure 9. It should be noted that a $\;t_{d2}$ of 4 μs results in poor performance being yielded when the tuning time ${\;t}_{t}$ is not zero in the analysis of Waveform 01, as reported in Section 3.2.1.

**Figure 10:**
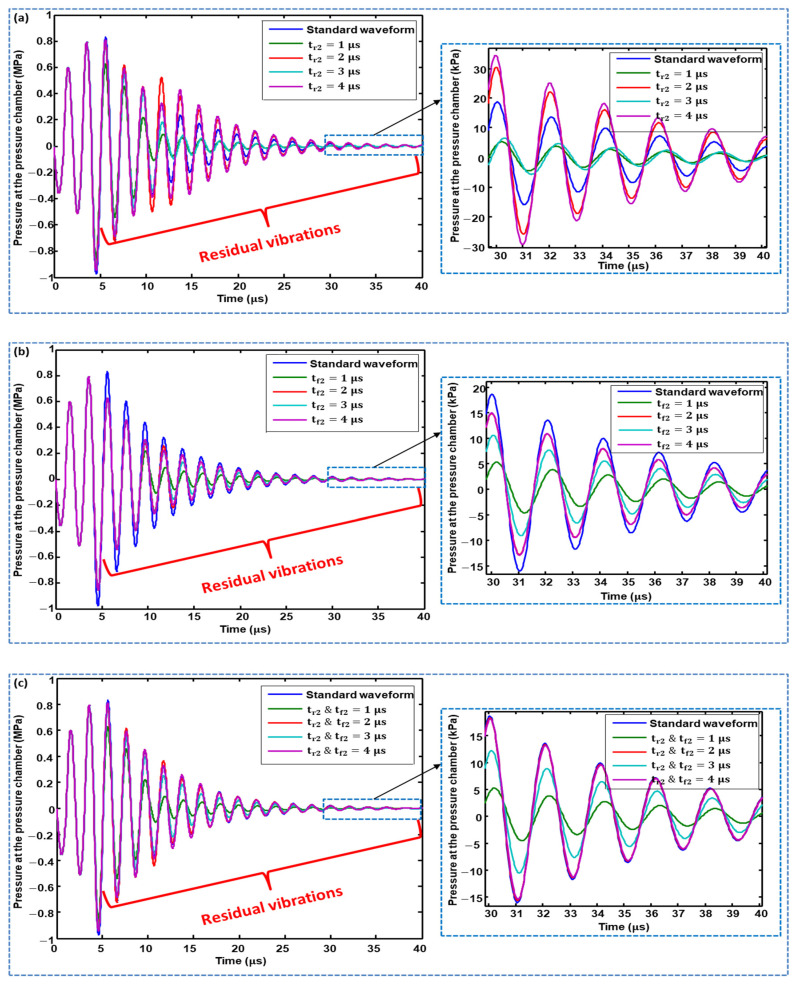
Effect of: (**a**) changing the rising time; (**b**) changing the falling time; and (**c**) simultaneously changing the rising and falling times of the second pulse of Waveform 02 on residual vibrations.

The effect of changing the rising and falling times of the second pulse on residual vibrations is shown in Figure 10, in which, when $\;t_{r2}=1\;\mathrm{and}\;3\;\mu s$, the amplitudes of residual vibrations are suppressed, while $\;t_{r2}=2\;\mathrm{and}\;4\;\mu s$ yields worse performance than even the standard waveform (see Figure 10a). In the case of ${\;t}_{f2}$, all values show suppressed amplitudes of residual vibrations, and $\;t_{f2}=1\;\mu s$ demonstrates the best performance, as shown in Figure 10b. Simultaneously changing $\;t_{r2}\;\mathrm{and}\;t_{f2\;}$ (Figure 10c) gives the same performance as that of Waveform 01. The optimized parameters for Waveform 02 are provided in Table 1.

#### 3.2.3. Voltage Amplitude Optimization of 2nd Pulse

**Figure 11:**
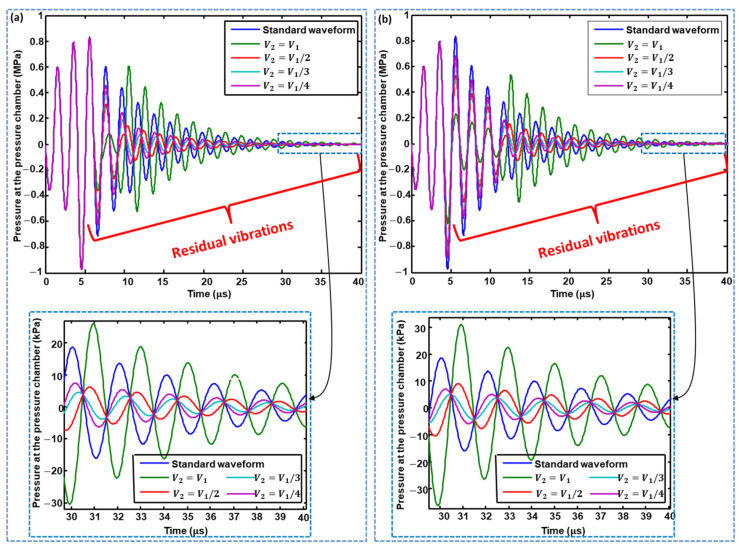
Effect of changing the voltage amplitude of the second pulse of: (**a**) Waveform 01; and (**b**) Waveform 02.

The voltage amplitude of the second pulse in both waveforms also affects the residual vibrations; therefore, it needs to be optimized. After optimization of the time intervals of the second pulse, the effects of changing the voltage amplitude of the second pulse on residual vibrations in the pressure profiles generated by Waveform 01 and Waveform 02 were analyzed and compared with those of the pressure profile generated using the standard waveform. Four voltage amplitudes were considered for the second pulse, i.e., the same amplitude, 1/3 the amplitude, and 1/4 the amplitude of the first pulse. As shown in Figure 11a,b, a voltage amplitude 1/3 times the amplitude of the first pulse gives the best performance concerning the suppression of residual vibrations for both Waveform 01 and Waveform 02, compared with the other three amplitudes. If the voltage amplitude of the second pulse is the same as that of the first pulse, then the performance becomes even worse than that of the standard waveform.

### 3.3. Comparison of Waveform 01 and Waveform 02

**Figure 12:**
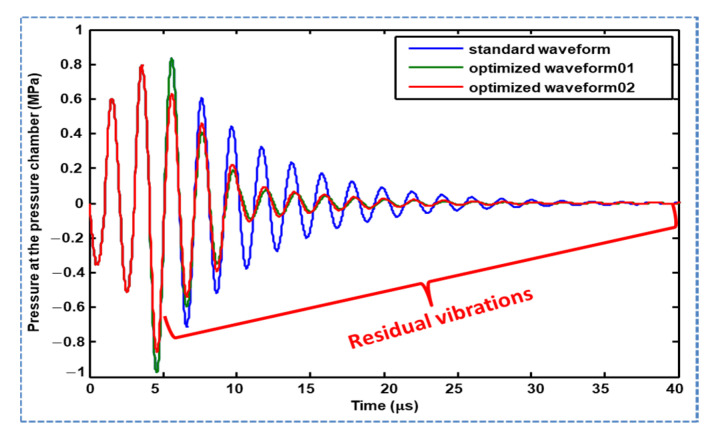
Comparison of Waveform 01 and Waveform 02 and their effect on residual vibrations.

**Figure 13:**
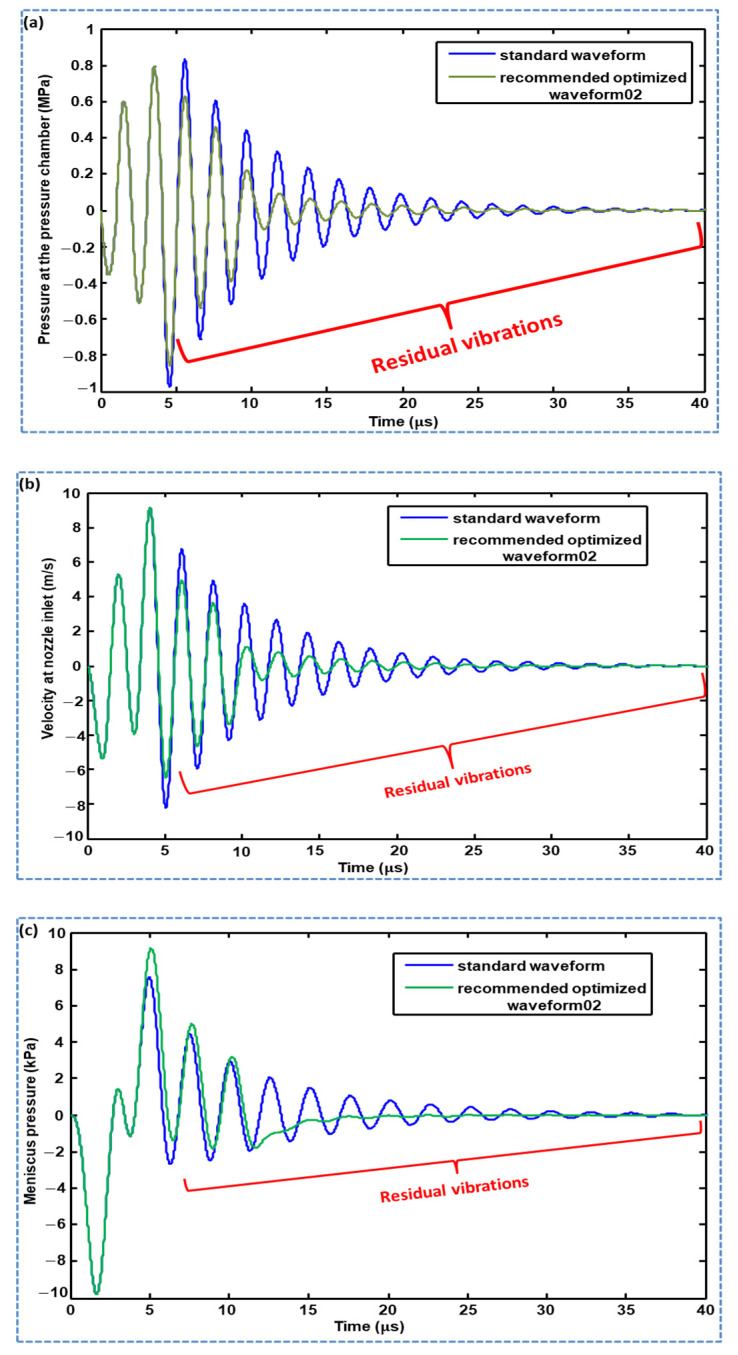
Comparison of profiles of: (**a**) pressure at the pressure chamber; (**b**) velocity at nozzle inlet; and (**c**) meniscus pressure generated by the standard waveform and recommended optimized Waveform 02.

A comparative analysis of the pressure profiles generated using Waveform 01 and Waveform 02 is reported in this section. Both waveforms successfully suppressed the residual vibrations compared with the pressure profile generated using the standard waveform, as shown in Figure 12. The suppression of residual vibration by the optimized Waveform 02 was greater than that by Waveform 01 in the time interval of 5–6 µs. Both optimized waveforms can be used to damp the residual vibrations, as both successfully suppressed the residual vibrations; however, in Waveform 01, the tuning time ($t_{t}$) is a sensitive parameter that gives even worse performance than the standard waveform if not optimized. In addition, two parameters must be optimized in the case of Waveform 01, that is, tuning time ($t_{t}$) and dwell time ($t_{d2}$); therefore, Waveform 02 is recommended for suppressing residual vibrations. Optimized parameters for both waveforms are presented in Table 1. The comparison of profiles of pressure at the pressure chamber, velocity at the nozzle inlet and meniscus pressure generated by the standard waveform and recommended optimized Waveform 02 are presented in Figure 13, which shows suppressed amplitudes of the residual vibrations by the recommended optimized Waveform 02.

## 4. Conclusions and Remarks

The lumped element model of a recirculating piezoelectric inkjet printhead was used to optimize the voltage waveforms for the suppression of residual vibrations. First, the standard voltage waveform was optimized for the case of maximum jetting pressure. However, the pressure profile acquired by the standard waveform has residual vibrations. To suppress these vibrations, two waveforms with optimized parameters were proposed, both of which successfully suppressed the amplitudes of the residual vibrations. The effects of changing the tuning time ($t_{t}$) and the rising time ($t_{r2}$), falling time ($t_{f2}$), and dwell time ($t_{d2}$) of the second pulse on the suppression of residual vibrations were analyzed. In the case of Waveform 01, $t_{t}$ and $t_{d2}$ of 2 μs yielded better performance than higher values of these parameters. In the case of Waveform 02, a $t_{d2}$ of 4 μs was the optimized value. The rising and falling times of the second pulse, $t_{r2}\;\mathrm{and}\;t_{f2}=1\;\mu s$ in Waveform 01 and Waveform 02, were found to be the optimal values. The odd values of both $t_{r2}\;\mathrm{and}\;t_{f2}$ provide better performance in suppressing residual vibrations compared with their even values. Furthermore, the voltage amplitude of the second pulse was also optimized; the optimal value was found to be 1/3 times the voltage amplitude of the first pulse. Some values of the three parameters, that is $t_{t}$ in Waveform 01, $t_{r2}$ in Waveform 02, and the voltage amplitude of the second pulse, led to worse performance if they were not optimized. Our analysis determined that the tuning time${\;t}_{t}$ in Waveform 01 is the most sensitive parameter. Therefore, Waveform 02 is recommended for use to suppress residual vibrations.

## Figures and Tables

**Table 1:** Optimized parameters of both waveforms for suppressing residual vibrations.

Waveform 01				Waveform 02 (Recommended)		
$\;t_{r1},\;t_{r2},\;t_{f1},\;t_{f2}$	$t_{d1}$	$t_{t}$	$t_{d2}$	$t_{r1},\;t_{r2},\;t_{f1},\;t_{f2}$	$t_{d1}$	$t_{d2}$
1 μs	2 μs	2 μs	2 μs	1 μs	2 μs	4 μs
